# Set-size effects for sampled shapes: experiments and model

**DOI:** 10.3389/fncom.2013.00067

**Published:** 2013-05-28

**Authors:** Christian Kempgens, Gunter Loffler, Harry S. Orbach

**Affiliations:** ^1^Fielmann Akademie Schloss PlönMeisterschule, Plön, Germany; ^2^Vision Sciences, Glasgow Caledonian UniversityGlasgow, Scotland

**Keywords:** sampled shapes, shape perception, shape model, set-size effect, visual search, orientation discrimination, heterogeneity detection, radial frequency patterns

## Abstract

The location of imperfections or heterogeneities in shapes and contours often correlates with points of interest in a visual scene. Investigating the detection of such heterogeneities provides clues as to the mechanisms processing simple shapes and contours. We determined set-size effects (e.g., sensitivity to single target detection as distractor number increases) for sampled contours to investigate how the visual system combines information across space. Stimuli were shapes sampled by oriented Gabor patches: circles and high-amplitude RF4 and RF8 radial frequency patterns with Gabor orientations tangential to the shape. Subjects had to detect a deviation in orientation of one element (“heterogeneity”). Heterogeneity detection sensitivity was measured for a range (7–40) of equally spaced (2.3–0.4°) elements. In a second condition, performance was measured when elements sampled a part of the shapes. We either varied partial contour length for a fixed (7) set-size, co-varying inter-element spacing, or set-size for a fixed spacing (0.7°), co-varying partial contour length. Surprisingly, set-size effects (poorer performance with more elements) are rarely seen. Set-size effects only occur for shapes containing concavities (RF4 and RF8) and when spacing is fixed. When elements are regularly spaced, detection performance improves with set-size for all shapes. When set-size is fixed and spacing varied, performance improves with decreasing spacing. Thus, when an increase in set-size and a decrease in spacing co-occur, the effect of spacing dominates, suggesting that inter-element spacing, not set-size, is the critical parameter for sampled shapes. We propose a model for the processing of simple shapes based on V4 curvature units with late noise, incorporating spacing, average shape curvature, and the number of segments with constant sign of curvature contained in the shape, which accurately accounts for our experimental results, making testable predictions for a variety of simple shapes.

## Introduction

Successful interaction with the environment requires the identification of the location of items of interest (Treisman and Gelade, 1980; Palmer et al., 2000), processing their shapes and textures (Field et al., 1993; Wilson and Wilkinson, 1998; Loffler, 2008) and recognizing objects (Biederman and Gerhardstein, 1993).

Points of interest catch the observer's attention, sometimes by a notch in a contour, a dent in a surface or by occlusion/superposition of items in the background by closer objects. Such points can be generalized as locations in a visual scene with some kind of deviation from the surround: a heterogeneity. Detection of such heterogeneities is crucial for the successful interpretation of our visual world.

As part of the ongoing research to investigate how the visual system accomplishes these tasks, the discrimination of—and search within—multi-element patterns has been employed widely. One particular focus has been on the effect of the number of elements in the display on sensitivity (set-size effect). The basic set size effect, whereby thresholds increase with set-size, has been explained on the basis of signal detection theory (Palmer et al., 1993, 2000; Palmer, 1994; Verghese and Nakayama, 1994; Parkes et al., 2001). While substantial set-size effects occur for uniform patterns (e.g., composed of elements with parallel orientation), it has recently been shown (Scott-Brown and Orbach, 1998; Kempgens et al., 2007) that signal detection theories of visual search cannot account for heterogeneous configurations (e.g., elements with random orientations; Orbach et al., 2005), where set-size performance is much worse than predicted. The difference in results for uniform vs. random configurations indicates that the orientational arrangement of pattern elements has a considerable effect on observer sensitivity.

In contrast to the heterogeneous conditions above, in this paper we will present data for heterogeneous cases where performance is actually better than what signal detection theories predict. This is the case when elements are positioned on the circumference of various contour shapes and therefore points to the crucial role of element arrangement. In line with other studies, the experimental results provide evidence for shape processing mechanisms (Wilkinson et al., 1998; Hess et al., 1999; Loffler et al., 2003; Poirier and Wilson, 2006), the outputs of which depend on a number of stimulus properties, such as element spacing (Field et al., 1993; Polat and Sagi, 1993; Levi and Klein, 2000), set-size, curvature (Field et al., 1993; Pettet, 1999; Loffler et al., 2003), and the complexity of the overall pattern shape (Pettet, 1999; Barenholtz and Feldman, 2003). The influence of several stimulus properties on shape processing can be explained with a model based on probability summation of the outputs of independent curvature detectors (Poirier and Wilson, 2006; Bell et al., 2009a), resulting in a population code for representing shapes (Pasupathy and Connor, 2002).

The objective of the experiments described below, was, first, to investigate the effect of set-size, shape, spacing and other factors on the detection of contour heterogeneity, second, to compare observers' performance to existing models of visual search, and third, since existing models fail, to devise a model that can account for the experimental results.

## Materials and methods

### Subjects

A total of nine subjects aged between 18 and 35 participated in the different experiments, usually three subjects per experiment, and all except one were naïve as to their purpose. All subjects had normal or corrected-to-normal acuity (≥6/6 with residual astigmatism ≤0.25D). Informed consent was obtained from each observer; and the study was approved by Glasgow Caledonian University's Life Sciences Ethics Committee. All experiments were conducted in accordance with the Declaration of Helsinki (WMA, 2008).

### General description of the stimuli

Stimulus patterns were pre-calculated and presented within the Matlab™ (The MathWorks) environment, supplemented by Psychophysics Toolbox extensions (Brainard, 1997; Pelli, 1997). Stimuli were composed of various numbers of high-contrast Gabor patches (Gabor, 1946) which were positioned on the circumference of simple shapes. The Gabor patches were defined by a two-dimensional Gaussian windowing a one-dimensional sinusoidal luminance grating. The phase of the Gabor's carrier grating was set to zero (cosine phase), and its contrast to −1, which resulted in a luminance minimum at the center of each element. The envelope's standard deviation was set to 0.085°, and the carrier grating spatial frequency to 7 cycles per degree (cpd).

The shapes that were sampled by the Gabors were radial frequency (RF) patterns (Equation 1). RF patterns (Wilkinson et al., 1998) are sinusoidally modulated circles where the shape is determined by the number of modulation cycles and by the amplitude of modulation.

(1) $$R(\varphi )=r_{0}\cdot (1+A\cdot \sin(RF\cdot \varphi +\theta ))$$

*R*(φ) is the radial distance of the contour from the center as a function of φ (polar angle), *r*_0_ is the size of the contour (radius of the unmodulated circle), A is the modulation amplitude, RF the radial frequency and θ the phase of the shape. The radial frequency determines the number of lobes of the RF pattern, the amplitude the “spikiness” of each lobe, and the phase the overall orientation.

**Figure 1:**
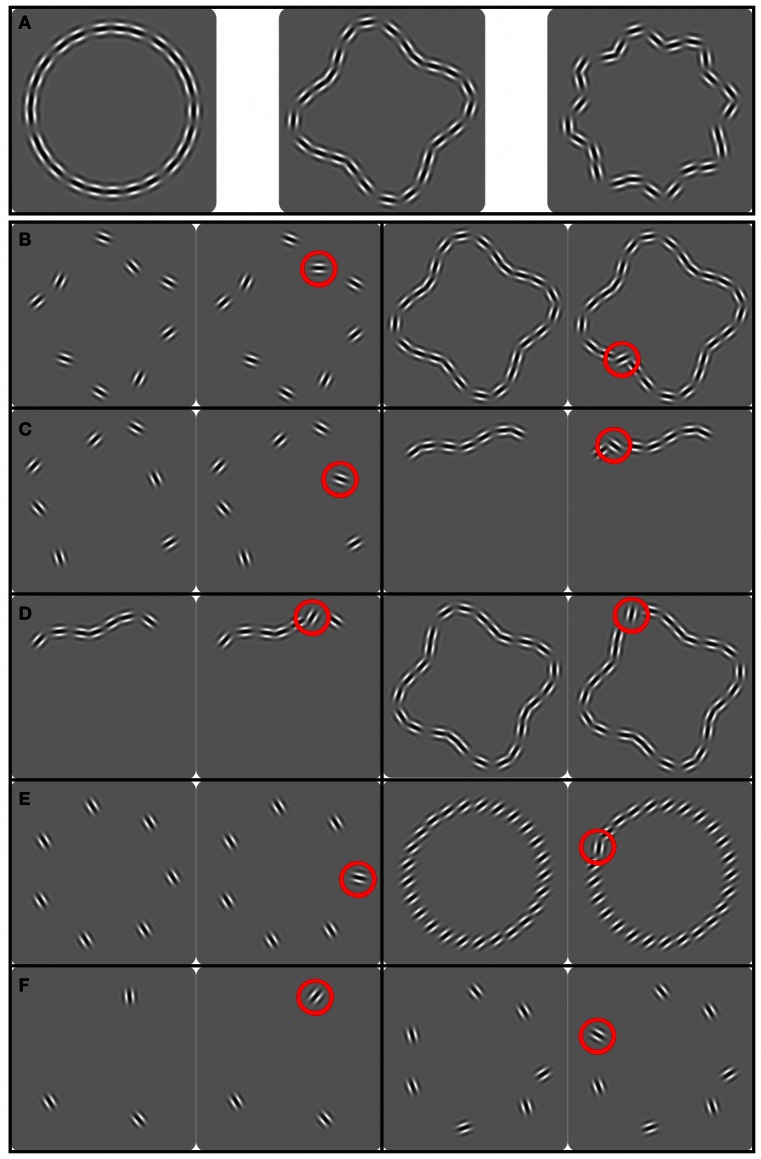
**Panel **(A)** shows the shapes used in the experiments (left to right) Circle, RF 4 and RF 8; panels (**B–F**) show the stimuli used in different experiments.** The two stimuli on the left are base and increment (circled red) pattern with low set-size [with wide spacing in panel **(C)**], the two stimuli on the right show the same with high set-size [with narrow spacing in panel **(C)**]. **(B–D)**: for reasons of brevity, the panels show examples of RF 4 shapes only, although all shapes shown in **(A)** were used; **(B)** shape alignment experiment, patterns ranging from sparsely to densely sampled entire shapes; **(C)** spacing experiment, patterns ranging from sparsely sampled entire shapes to short, densely sampled partial shapes; **(D)** set-size experiment, patterns ranging from short, densely sampled partial shapes to equally dense closed contours; **(E)** uniform orientation experiment, parallel Gabors sampling a circle; **(F)** random orientation experiment, randomly oriented Gabors sampling a circle.

We used three different RF shapes [see Figure 1, panel **(A)**]: a circle (RF 0 and *A*_RF0_ = 0), a “dented square” (RF 4 and *A*_RF4_ = 0.18) and a “rounded star” (RF 8 and *A*_RF8_ = 0.1). The amplitude of the RF 4 and 8 patterns were chosen so that the resulting shapes contained both obvious convexities and concavities. In different conditions, the orientations of the Gabor patches were either parallel to each other, random or tangential to the sampled contour. For each shape, the number of Gabors sampling the contour (set-size) was varied. The mean radius of all contours was set to *r*_0_ = 2.7°. To avoid learning effects, global rotations of the entire pattern were effected by setting the pattern angular phase to 100°, 111.25°, or 122.5°, placing the lobes of RF 4 and RF 8 patterns at three different angles.

Pilot experiments (Kempgens et al., 2006) showed that, depending on the shape and number of elements, some configurations exhibited a bilateral symmetry, which could be used by subjects. To avoid such cues being present in some but not other conditions, the position of each element was subject to a small amount of random jitter along the contour by a polar angle δ_i_, according to Equation 2, where δ_max_ is the maximum jitter, *n* is the number of pattern elements, *i* is the patch number, and *rand* is a random number (uniform distribution) between 0 and 1.

(2) $$\delta_{i}=\delta_{\max}\cdot rand\text{ with }\delta_{\max}=0.4\cdot \frac{360^{{}^{\circ}}}{n}-3^{{}^{\circ}}$$

This avoids symmetry but ensures sufficient inter-element spacing (center-to-center spacing) so that adjacent patches for patterns with large numbers of elements are not overlapping. In addition, randomizing element position (and therefore inter-element spacing) prevents observers from building an internal template for any condition.

### Procedure

For most experiments, a temporal 2AFC procedure with the method of constant stimuli was used, where base and increment patterns were presented successively in random order. In a base pattern, all elements were either parallel to each other (Figure 1E) or aligned to the shape contour (Figures 1B–D). In an increment pattern, the orientation of one Gabor patch deviated from this orientation (either not parallel to the other elements or oriented away from being tangential to the shape). The task was to indicate which of the two stimuli contained the heterogeneous Gabor by pressing one of two keyboard buttons. No feedback was given.

The first stimulus was preceded by a blank pre-stimulus interval of 400 ms, where the monitor was set to a uniform mid-gray. Each stimulus was shown for 200 ms, and stimuli were separated by a 1000 ms blank screen. A central fixation dot was present throughout. Different conditions (shape, number of elements) were presented in different blocks. For each condition, six levels of orientation increments were used, identical for all observers. The magnitude of the increments was different for different conditions and selected according to pilot experiments. Subjects were given 20 practice trials before each condition and the shape sampled by the Gabor patches was shown as a continuous contour prior to the experimental runs. Each increment was presented twenty times in random order and subjects completed two runs for each experimental condition, resulting in 240 trials per condition. Performance was measured in terms of percentage correct as a function of orientation increment. Data were fit with a Weibull function (Weibull, 1951) using a maximum likelihood procedure (Watson, 1979) and thresholds defined as the orientation increment yielding a 75% correct performance.

### Apparatus

A Philips monochrome CRT monitor, set to its low, one-gun, mode with 256 gray levels, a frame refresh rate of 85 Hz, and a spatial resolution of 1024 × 768 pixels (pixel width = 0.0177° at 1.2 m viewing distance), was used to display the stimuli. In order to avoid horizontal and vertical orientation cues from the monitor frame, a white cardboard mask with a 19.5 cm diameter circular aperture (9.3° visual angle) was placed in front of the monitor. The mask was illuminated by four tungsten light bulbs in order to match the luminance on the mask to the mean luminance of the screen (138 cd/m^2^). The monitor was linearized by adjusting its look-up table, resulting in 151 approximately equally spaced gray levels (Pelli and Zhang, 1991; Brainard et al., 2002). Subjects viewed stimuli binocularly at a distance of 1.2 m. A constant distance was maintained by using a chin and forehead rest.

### Data analysis

Observer performances were averaged and orientation increment thresholds plotted as a function of set-size in log-log coordinates. The data were fit with power functions, which, in log-log coordinates, result in straight lines and their slope is a measure of the set-size effect (Palmer, 1994). Univariate Analysis of Variance (ANOVA) within SPSS (IBM) was performed on the data to test for significant effects of set-size and shape. *Post-hoc* tests (Bonferroni corrected paired t-tests) established whether thresholds for different shapes were significantly different for individual set-sizes.

## Results

### Uniform orientation experiment

The aim of the first experiment was to determine the effect of set-size when elements are positioned on a circle and share the same orientation. A set of 3, 9, 13, 17, and 25 uniform (i.e., parallel) Gabors were positioned on a circle, with orientations constant within a trial but randomly varied between trials (Figure 1E). The task was to indicate which of two intervals contained the stimulus where the orientation of one element deviated from being parallel to the other elements.

**Figure 2:**
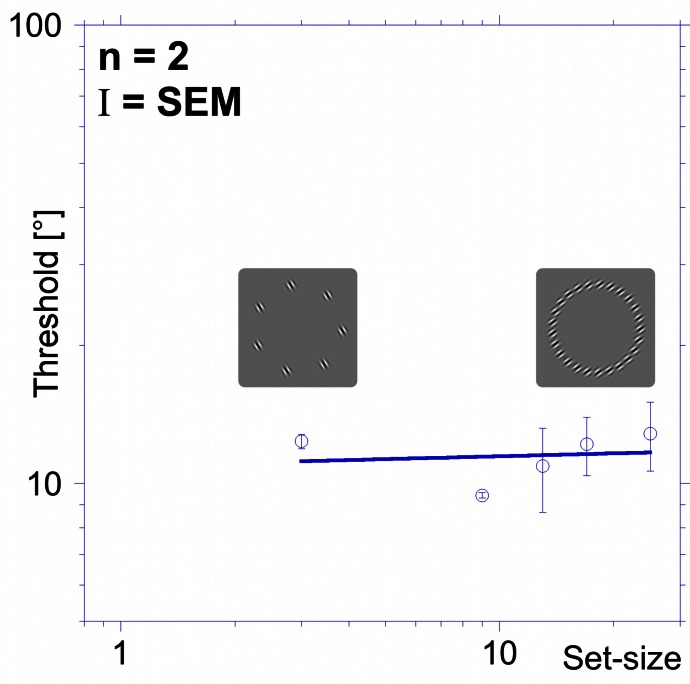
**Threshold vs. set-size for parallel elements (uniform orientation experiment).** Average thresholds (orientation change for one element required to detect it within a variable number of parallel elements) for two subjects are plotted as a function of set-size (number of pattern elements). Sensitivity is largely independent of element numbers, evidenced by the flat set-size slope (+0.02). Error bars here and elsewhere are standard error of the mean (SEM). The icons show two examples for the uniform stimuli with a set-size of 7 (left) and 25 (right) elements.

The results of the “uniform-orientation” experiment are presented in Figure 2. Average thresholds are displayed as a function of set-size. Thresholds for detecting the orientation change of one element range from 9.4° to 12.9° for set-sizes between 3 and 25. The slope of the set-size function (*S*_uniform_ = +0.02), is essentially flat, indicating that set-size does not affect performance. Hence, contrary to predictions from standard models of visual search, sensitivity does not decrease with increasing set-size when elements have uniform orientations.

### Shape-alignment experiment

Standard models of visual search, employing a max decision rule, predict performance to decrease with increasing number of elements with a magnitude given by the slope of the set-size vs. threshold function (set-size effect). These models predict a slope between 0.20 and 0.35 when elements are sufficiently separated (Palmer, 1994). The flat set-size slope for the uniform orientation condition (Figure 2) is inconsistent with this prediction.

This raises the question of whether there are other configurations, where element orientations are non-parallel that would also show performance independent of set-size. One possibility is to place elements on the circumference of closed contour shapes with orientations tangential to the sampled shape (“shape alignment,” see Figure 1B). Employing a variety of pattern shapes allowed us to determine the generality of any effect and to provide insight into possible shape encoding processes.

The shapes from which elements were sampled were RF patterns with radial frequencies of 0, 4 and 8 (circular, “dented square” and “rounded star” shapes). Set-sizes used were 3, 7, 9, 13, 17, 25, and 40, except for the RF 8 where set-sizes 3 and 7 were omitted because a pilot study showed that threshold to detect the orientation change of one element was above the maximum change of 90° that could be applied. The task was to identify the interval in which the orientation of one Gabor was misaligned with the contour shape.

**Figure 3:**
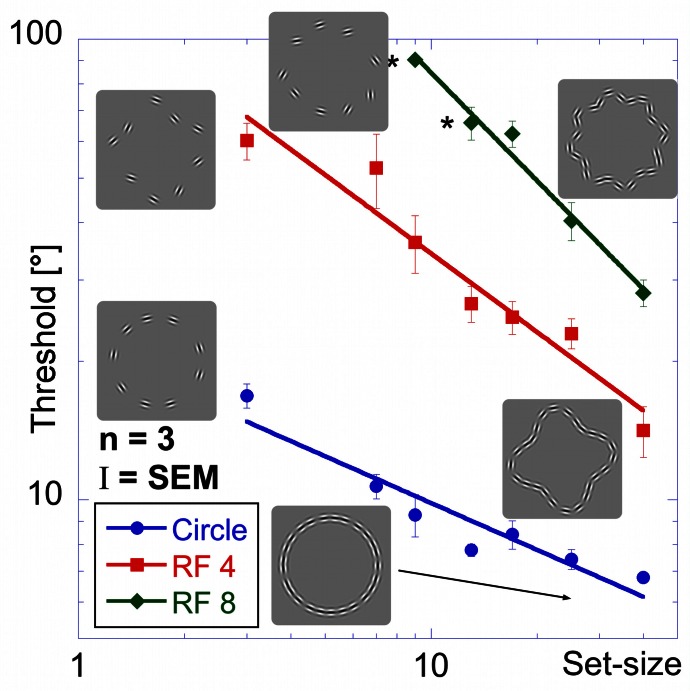
**Shape alignment experiment.** Average performance for detecting the misalignment of one element sampled from a circle (blue circles), RF 4 (red squares) and RF 8 (green diamonds, shapes shown by icons) as a function of the total number of samples (set-size). Thresholds are given as the orientation deviation of one element required to discriminate the pattern with the misaligned element against a pattern where all elements were tangential. Asterisks indicate set-sizes where not all observers were able to reach threshold performance.

The results of the “shape alignment” experiment can be seen in Figure 3. Performance shows a clear dependence on set size with thresholds ranging from 7 to 17° for the circular shape (blue, circular symbols in Figure 3), from 14 to 60° for the RF 4 (red squares), and from 28 to about 90° for the RF 8 (green diamonds).

Data were analysed using an ANOVA with shape and set-size as factors. This analysis found significant main effects for shape [*F*_(2, 57)_ = 294.43, *p* < 0.025] and set-size [*F*_(6, 57)_ = 31.59, *p* < 0.025]. The data also show a significant interaction between shape and set-size [*F*_(10, 57)_ = 12.47, *p* < 0.025], reflecting the difference in set-size slopes for the three shapes. Pair-wise comparisons showed significant (*p* < 0.025) differences for corresponding set-sizes between each of the shapes tested.

Contrary to standard models of visual search, thresholds here improved with set-size for each shape. This is an example of an *inverse* set-size effect with negative slopes of −0.34, −0.57, and −0.78 for circle, RF4 and RF8, respectively. Slopes for different shapes are significantly different from each other (*p* < 0.025).

### Random orientation experiment

The first two experiments showed a flat set-size slope for detecting an orientation increment for uniformly oriented elements and a negative slope for elements aligned along a contour. The aim of this experiment was to devise a baseline for these performances by determining the set-size effect for patterns with elements positioned as before (on a circle), but with random orientations.

**Figure 4:**
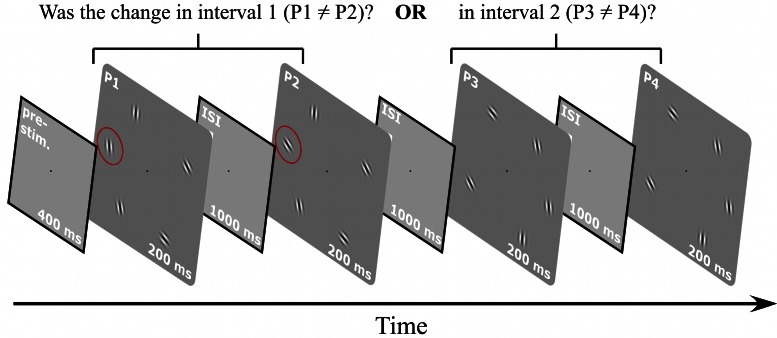
**Procedure for random orientation experiment.** A 400 ms pre-stimulus interval was followed by two intervals containing two stimuli each (i.e., interval one with patterns P1 and P2, interval two with patterns P3 and P4). The orientation of one element in one interval changed so that either P1 and P2 or P3 and P4 were not identical. The task was to indicate which interval contained the non-matching stimuli. The orientations of the elements for each trial and each interval were randomly selected. Stimuli were presented for 200 ms, separated by 1000 ms inter-stimulus intervals. A blank screen was shown until the subject responded.

The increment patch in a uniform pattern stands out because it deviates from a common feature (uniform orientation) of all other patches. This is not the case for randomly oriented elements, so the labels base and increment pattern are ambiguous, making the task of detecting a Gabor patch with an orientation increment impossible in a standard 2AFC paradigm. Therefore, the stimulus sequence and task had to be modified in this experiment, as shown in Figure 4. Each trial of the modified 2AFC consisted of two intervals where each interval contained two sequentially presented stimuli (pattern pairs). The task was to indicate which interval contained the pattern pair that was not identical, i.e., where the orientation of one element differed between the two stimuli. Set-sizes were *N* = 3, 5, and 7. The base patterns consisted of *N* Gabors with randomly assigned orientations (Figure 1F). A different base pattern was presented on each trial. The location of the patch that changed in orientation was varied randomly from trial to trial. Orientation increments were randomly chosen to be either clockwise or anti-clockwise.

**Figure 5:**
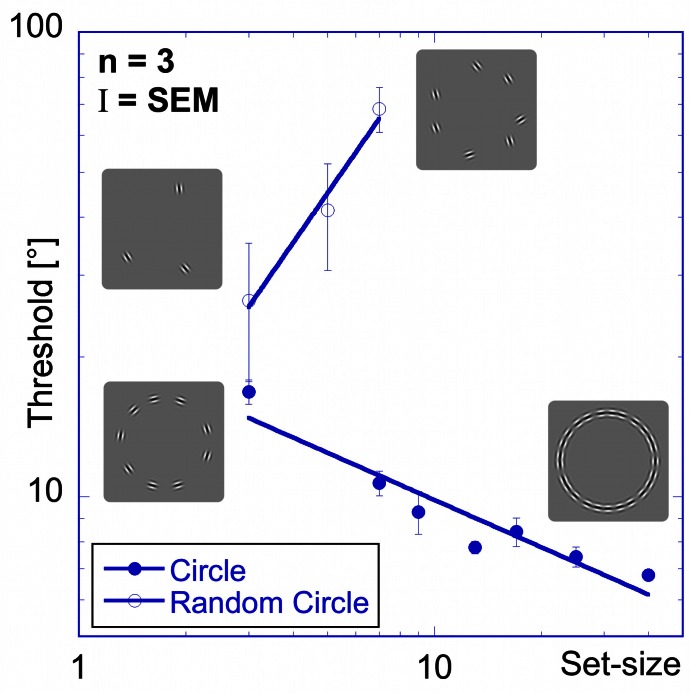
**Random orientation experiment.** Comparison of performance for patches aligned to a circle (filled circles) and randomly oriented patches on a circle (open circles) shows a strong positive set-size slope for the latter, in sharp contrast to the negative set-size slope for the former. The insets show examples of the stimuli.

The random orientation experiment shows that a change in orientation of one element, when embedded in a configuration of elements with random orientations, becomes increasingly harder to detect the more elements there are in the pattern. Figure 5 shows thresholds for detecting a change in orientation as a function of set-size. In contrast to the thresholds for the “aligned circle” (Figure 3), the data for the random orientation experiment show a strong positive set-size effect (slope = +1.1), confirming earlier results (Orbach et al., 2005).

### Spacing experiment

In the “shape alignment” experiment (Figure 1B), the spacing between pattern elements co-varied with set-size. To isolate the influence of inter-element spacing, elements in this experiment were also positioned on RF shapes but set-size was kept constant (seven elements) while the spacing between elements was varied. Reducing the average spacing of seven Gabors resulted in patterns ranging from complete, but sparsely sampled, RF shapes to short, but densely sampled, partial RF shapes (Figure 1C).

**Figure 6:**
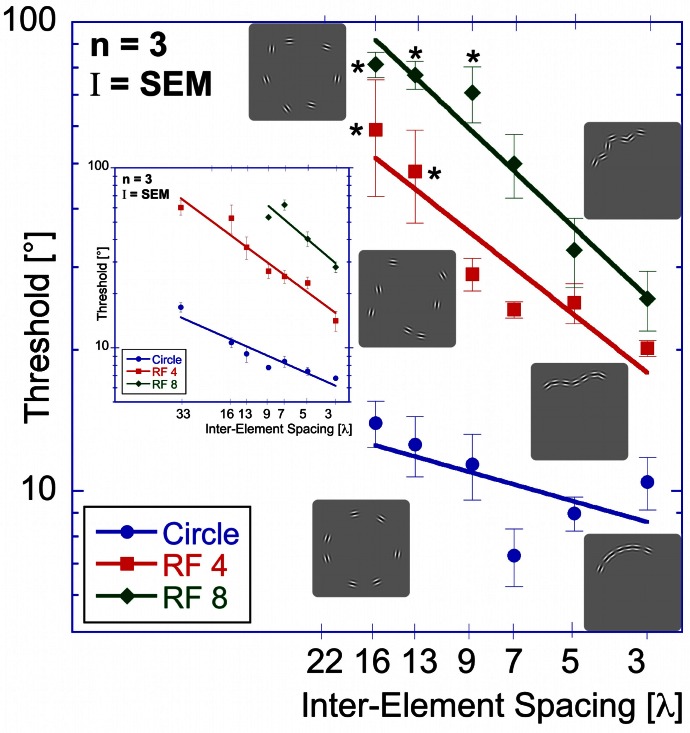
**Spacing experiment.** Average performance (three observers) for circular (blue circles), RF 4 (red squares) and RF 8 (green diamonds) is displayed as a function of inter-element spacing expressed as multiples of the Gabor carrier wavelength (λ). Asterisks indicate conditions where the threshold of at least one observer was above the maximum of 90° and the resulting value is the mean across the remaining observers. The inset shows the results of the “shape alignment experiment” as a function of inter-element spacing. It is immediately obvious that the results of shape alignment and spacing experiments are very similar, and that in both cases thresholds decrease with decreasing inter-element spacing.

Thresholds were determined for a circle, RF 4, and RF 8 (using the standard 2AFC procedure) and for the random orientation condition (modified 2AFC procedure). Data are presented as a function of inter-element spacing, which was calculated as the average distance between two adjacent elements (Figure 6). Six different spacings were tested (0.4, 0.7, 1.0, 1.3, 1.8, and 2.3° corresponding to 3, 5, 7, 9, 13, and 16 λ, where λ is the Gabor carrier wavelength).

Thresholds for circular shape range between 14° (for the widest spacing, 16 λ) and 7.3° (7 λ). For the RF 4, thresholds range from 59° (16 λ) to 20° (3 λ) and from 81° (16 λ) to 26° (3λ) for RF 8. Statistical analysis showed significant main effects for shape [*F*_(2, 51)_ = 223.52, *p* < 0.025] and spacing [*F*_(5,51)_ = 35.23, *p* < 0.025] and a significant interaction between them [*F*_(9, 51)_ = 13.31, *p* < 0.025], consistent with the different slopes. *Post-hoc* tests showed significant (*p* < 0.025) differences for corresponding spacings between each of the three shapes tested, with the exception of RF 4 vs. RF 8 with 3, 5, and 13 λ spacing (*p* = 0.127, 0.72, and 0.063). The slopes were −0.22, −0.6, and −0.72 for circle, RF4 and RF8, respectively. The slope for the circle was significantly different from those for RF 4 and 8 (*p* < 0.025), but the slopes for RF4 and RF8 were not (*p* = 0.378).

There is an obvious similarity between the data for the “shape alignment” experiment (Figure 3) and the “spacing” experiment (Figure 6) when data are plotted with decreasing inter-element spacing on the abscissa. To quantify this, we conducted an ANOVA with the experimental condition (shape alignment vs. spacing experiment) as an additional factor. This analysis revealed no main effect of experimental condition [*F*_(1, 114)_ = 0.145, *p* = 0.704], suggesting that co-varying the number of elements with inter-element spacing seems to have no effect on thresholds.

Performance was also measured for the random orientation condition as a function of inter-element spacing, using the modified procedure described above, although the number of elements was reduced to five patches because observers could not complete the task with seven randomly oriented elements. Seven inter-element spacings were tested (the same as above plus one additional of 3.2° or 22 λ). This additional spacing corresponds to the spacing between five equally spaced Gabor patches.

**Figure 7:**
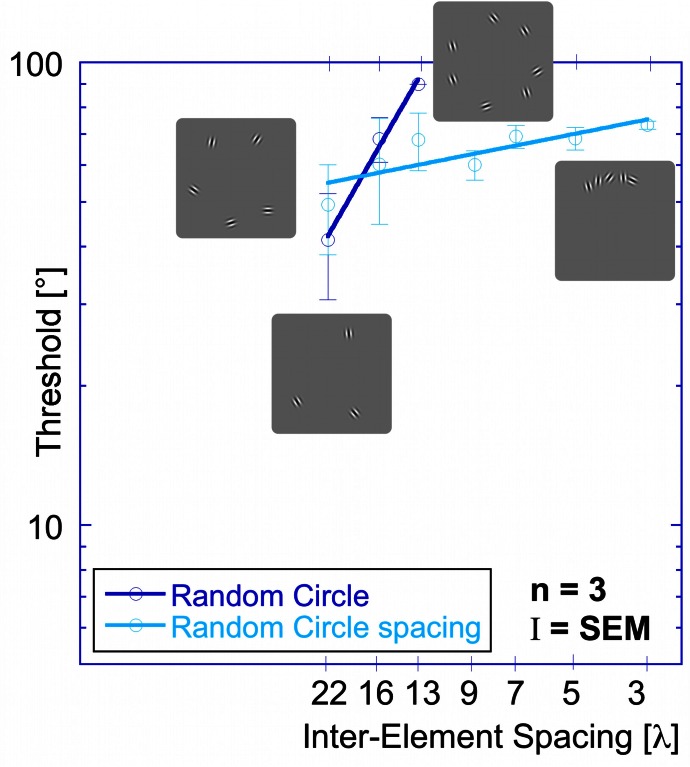
**Comparison of thresholds as a function of inter-element spacing for the random orientation experiment (dark blue circles) and the random orientation spacing experiment (light blue circles).** (Note that the thresholds for the random orientation experiment are different from the ones shown in Figure 5 because they come from different subjects. Importantly, for both subject groups, the slopes are similar (≥1) and the qualitative results are the same.)

Performance is considerably worse for randomly oriented elements (Figure 7) than for aligned elements (Figure 6). Thresholds for the random condition slightly increase with decreasing inter-element spacing. This is in contrast to the data for the random condition as a function of element numbers (Figure 7), where thresholds rose dramatically with set size. Hence, the similarity between the data for set-size and inter-element spacing that is evident when elements are aligned to contour shapes is absent when they are oriented randomly.

### Set-size experiment

The final experiment was designed to investigate the effect of set-size independent of inter-element spacing. Inter-element spacing in circular, RF 4 and RF 8 shapes was kept constant at 5 λ. This spacing corresponds to the relatively narrow spacing of the 25-element patterns in the “shape alignment” experiment. Six set-sizes (3, 7, 9, 13, 17, and 25 elements) were tested, resulting in stimuli ranging from very short, densely sampled partial contours to equally densely sampled circular, RF 4 or RF 8 contours (Figure 1D).

**Figure 8:**
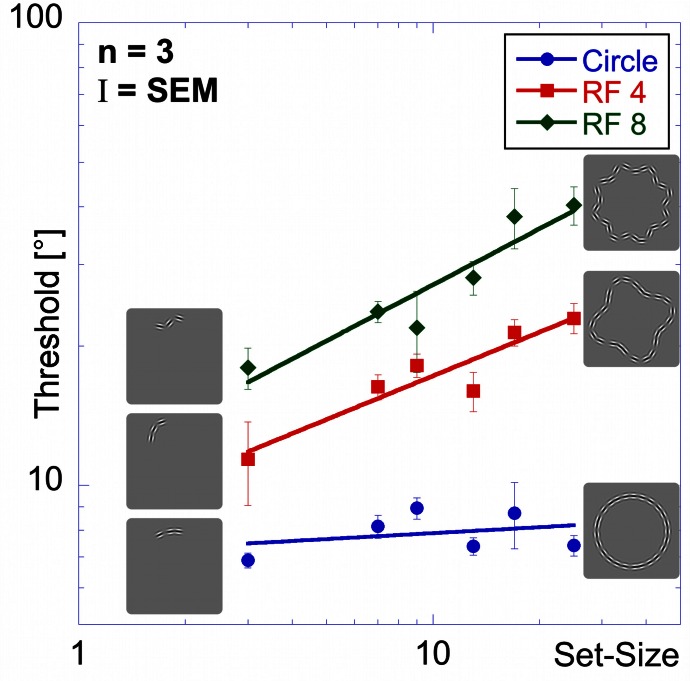
**Set-size experiment.** Stimuli in the set-size experiment had constant inter-element spacing. Average thresholds (three observers) are shown as a function of set-size for circular contours (blue circular data points), RF 4 (red squares) and RF 8 (green diamonds). Insets show the arrangement for patterns of 3 elements (left) and 25 elements (right). Performance is best for the circle, followed by the RF4 and worst for the RF8. The set-size slope is almost zero for circular patterns, and significantly higher for RF 4 and RF 8.

As in previous experiments, performance is best for the circle, intermediate for the RF 4 and worst for the RF 8 (Figure 8).

Thresholds for the circle range between 6.9 and 8.9° and are largely unaffected by set-size, resulting in a flat set-size curve (slope = +0.04). This shows that, with constant spacing, processing of circular patterns is independent of the number of pattern elements. Mean thresholds for the RF 4 increase from 11.4° for 3 elements to 23.0° for 25 elements. The set-size effect shows a positive slope of 0.31. For the RF 8 shape, a moderate set-size effect was also found (slope = +0.40), with thresholds increasing from 18.0 to 40.3°.

Statistical analysis reveals main effects for shape [*F*_(2, 54)_ = 140.80, *p* < 0.025] and set-size [*F*_(5, 54)_ = 12.91, *p* < 0.025] and a significant interaction [*F*_(10, 54)_ = 4.75, *p* < 0.025], corresponding to the different slopes. *Post-hoc* tests show that for the same set-size, thresholds for circular partial contours are significantly lower than for RF 4 and RF 8 and thresholds for RF 4 partial contours typically lower than for RF 8 (*p* < 0.025, with the exceptions of 3- and 7-element RF 4 vs. circle and RF 8; and 9-and 13-element RF 4 vs. RF 8, which are not significant). Furthermore, the set-size slope for circular patterns is significantly lower than for RF 4 and RF 8 patterns (*p* < 0.025), whereas the slopes of RF 4 and RF 8 are not significantly different (*p* = 0.548).

It is interesting that our data do not show an advantage of closure (Kovacs and Julesz, 1993). Comparing thresholds for the closed conditions (25 element) with those for the open partial contours did not lead to a significant improvement in performance for any of the shapes and actually caused a decrease in performance for RF 4 and RF 8.

## Model for shape processing

This section describes a general model for shape processing that we applied to our data for observers' ability to detect an orientation change of one element in sampled circles and RF shapes. It is based on physiologically plausible models previously described in the literature (Wilson et al., 1997; Achtman et al., 2003; Poirier and Wilson, 2006) but contains substantial modifications, which are critical for its success in dealing with sampled shapes containing convexities and concavities.

**Figure 9:**
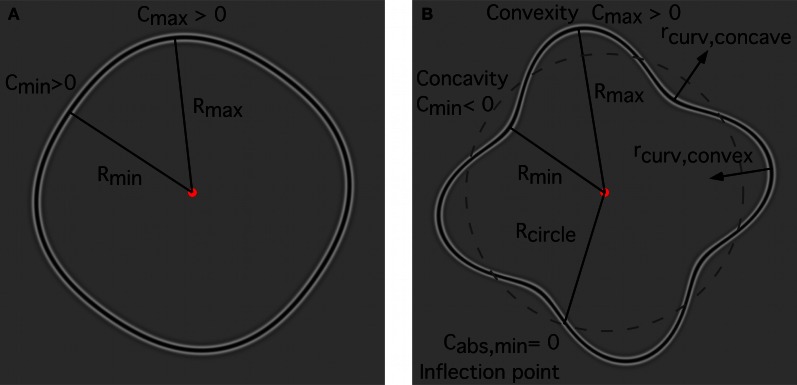
**Local curvature for RF contours. (A)** low amplitude RF4; **(B)** high amplitude RF 4. Regardless of amplitude, curvature minima (*C*_min_) for RF shapes are located at the points where the pattern radius is at a minimum (*R*_min_) whereas curvature maxima (*C*_max_) are at the maximum radius (*R*_max_). **(A)** for a sufficiently low amplitude, the local curvature of an RF shape is exclusively convex (curvature > 0) and contains no inflection points where curvature changes sign. Consequently, these shapes consist of only one segment of positive curvature (or one arc); **(B)** for a sufficiently high-amplitude, curvature changes from convex to concave and this change of curvature sign occurs at inflection points. For an RF N shape, there are 2N inflection points separating 2N arcs of alternating sign of curvature. The minimum (unsigned) curvature (*C*_abs,min_) is zero, and is located at the intersections of the RF contour with the unmodulated circle (dashed contour). The (signed) curvature is positive for convex contour parts and negative for concave parts. The local radius of curvature for convexities (*r*_curv,convex_) points toward the interior of the RF shape; the local radius of curvature for concavities (*r*_curv,concave_) points away from the shape's center.

Model responses are determined by stimulus characteristics such as inter-element spacing, curvature and complexity of the shape, and are based on probability summation of independent mechanisms. One of the important model features concerns the local curvature of a shape and how it changes along the contour. Circles and RF patterns with sufficiently low amplitude are exclusively convex contours (Figure 9A). In RF *N* patterns (*N* = radial frequency) with a sufficiently high amplitude (Figure 9B), curvature changes sign at 2N inflection points, separating 2N segments that are alternatingly convex and concave, i.e., alternating segments with positive and negative curvature. In the following text segments without changes of the sign of curvature will be termed “arcs.” For the complete contours used in the “shape alignment” experiment, the circle consists of only one arc, the RF 4 of eight arcs, and the RF 8 of sixteen arcs. For the incomplete shapes used in the “spacing” and “set-size” experiments, the number of arcs depends on the fraction of the shape that is sampled (see below). As for curvature magnitude, curvature minima (*C*_min_) are located where the pattern radius is at a minimum (*R*_min_), and curvature maxima (*C*_max_) at the maximum radius (*R*_max_). At the intersections of the RF contour with the unmodulated circle (dashed contour, Figure 9B), unsigned curvature (*C*_abs,min_) is at a minimum and, for sufficiently large amplitudes as used here, is equal to zero.

### Description of the model

#### Stages 1–3: contour, center, and size

Based on a biologically plausible model for processing RF contours (Poirier and Wilson, 2006), our model consists of five stages. The first stage extracts local contour information, which serves as input to successive stages of the model. From this, stage 2 computes the center of the RF pattern (Wilson et al., 1997; Wilson, 1999; Poirier and Wilson, 2006), and, stage 3, its size. The pattern size is required to determine the distance from the RF center to the locations where subsequent processing, i.e., extraction of curvature, takes place. For a detailed description of these first three stages the reader is referred to Poirier and Wilson (2006).

#### Stage 4: extraction of local curvature

The original Poirier and Wilson model (Poirier and Wilson, 2006) only directly describes shape processing for *convex* RF patterns because it contains a half-wave rectification of the outputs of curvature detectors which restricts its computation to isolating points of convex curvature maxima only, neglecting concave curvature. This is consistent with experimental results (Loffler et al., 2003) showing that RF discrimination sensitivity is affected most strongly when points of convex curvature maxima are occluded, although other parts of the contour have been shown to play a non-trivial role (Poirier and Wilson, 2007; Hancock and Peirce, 2008; Bell et al., 2010). These observations were, however, for RFs with low amplitude, which do not contain concavities. In our experiments, the RF 4 and RF 8 patterns were of sufficiently high amplitude so that patterns contained concavities. It has been shown (Pettet, 1999) that observers are better at detecting a contour of Gabor patches in noise when the contour does not change the sign of curvature. It is, therefore, possible that the different results in our experiments for circular and non-circular RFs are related to contours either containing exclusively convex curvature (circle) or containing changes in the sign of the curvature (RF4 and 8). In sum, this suggests that, in some experimental conditions, curvature information from concavities is required to explain performance. We therefore modified Poirier and Wilson's model to respond to local points of convex and concave curvature and this allows the model to be successfully applied to our results.

**Figure 10:**
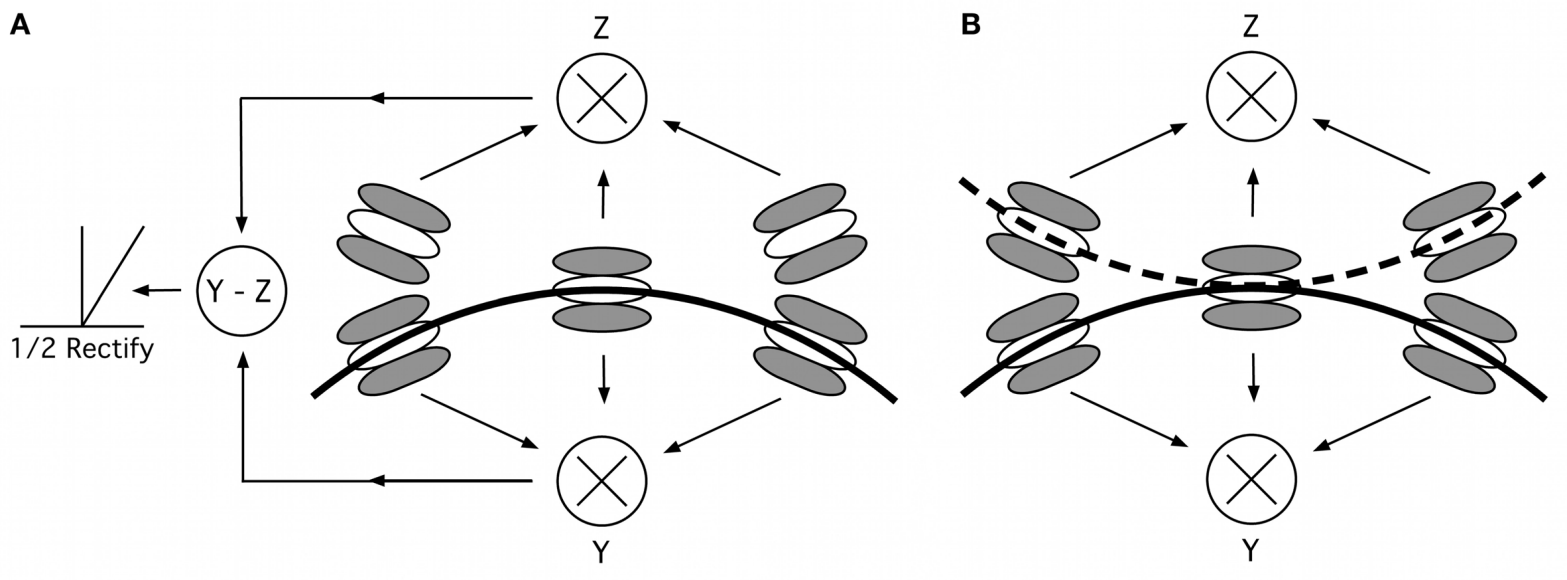
**Curvature mechanisms. (A)** Mechanism suggested by Poirier and Wilson (2006), consisting of five oriented filters, arranged along two curved lines of opposite curvature. The responses for each of two filter triplets (center plus two filters above or below the midline) are multiplied and their responses subtracted and half-wave rectified, to isolate points of convex curvature. **(B)** Removing the subtraction and rectification stage allows our modified curvature mechanism to process both convexities and concavities. The solid curve optimally stimulates the lower triplet “Y”; the dashed curve stimulates the upper triplet “Z.” Depending on the location of the overall contour's center, the response reflects convex or concave curvatures.

Stage 4 of our model consists of multiple local curvature units, responding to different points along the contour (as determined by the earlier stages). Local curvature units sample information from multiple orientation detectors (Figures 10A,B; Poirier and Wilson, 2006). For each curvature unit, five orientation selective filters, all tuned to the same spatial frequency, are arranged in such a way that they fall along the paths of two circular arcs of opposite sign of curvature. One central filter is located at the point where the two arcs touch; the other filters are positioned on the two arcs, equidistant on either side of the central filter with orientation preference tangential to the arcs (Figure 10B). Depending on the filters' spatial frequency tuning, their relative locations and orientation preference, curvature units can be made to respond to a multitude of combinations of spatial frequencies and curvatures. To enable the model to respond to a range of curvatures with different profiles, additional curvature units are implemented with filters tuned to different spatial frequencies and with different relative distances and angles between central and peripheral filters. Competitive interactions between curvature units, each tuned to a combination of spatial frequency/profile and curvature, via mutually inhibitive connections, results in the most highly active unit driving the model response. An anatomical candidate for this is V2, which has been shown to respond to curved stimuli (Hegdé and Van Essen, 2000), lying between V1, where we assume local contour information to be encoded, and V4, where population codes for shape are formed (Pasupathy and Connor, 2002).

When processing a contour, only three of the five filters, a triplet, will typically be excited. Their responses are multiplied, an operation which ensures that when one of the three filters is not sufficiently stimulated (e.g., if the curvature of the contour segment is different from the curvature to which the curvature mechanism is tuned or, in our experiments, by an increment patch with orientation that sufficiently deviates from tangential), the output of the filter triplet is small or zero. The optimal stimulus for the three filters, yielding maximum response, is a contour, which passes through the three filters tangential to the filters' orientation (indicated by the solid curved line in Figure 10).

In Poirier and Wilson's model, the outputs of the two filter triplets are subtracted and the result is half-wave rectified (Figure 10A), which results in activation of only one of the two filter triplets (in this case Y but not Z). This restricts the computation on e.g., convex curvature maxima. In our modification, the responses of the two filter triplets are not subtracted and not half-wave rectified, so that curvature information is available for both convexities (unit Y, assuming a global pattern center that lies below the contour) and concavities (unit Z).

#### Stage 5: population code of arc units with late noise as a representation of shape

The results of the shape alignment experiment (Figure 3), the spacing experiment (Figure 6) and the set-size experiment (Figure 8) are inconsistent with standard models for visual search (Palmer et al., 2000). These models assume early noise where performance is limited by the noise of units low in the hierarchy of visual processing, e.g., V1 cells. The overall performance in the presence of multiple elements is based on a probabilistic summation of the responses of individual units. Increasing the number of distractors while keeping the number of signal elements constant increases the total amount of noise and predicts an increase in threshold in line with positive set-size slopes.

**Figure 11:**
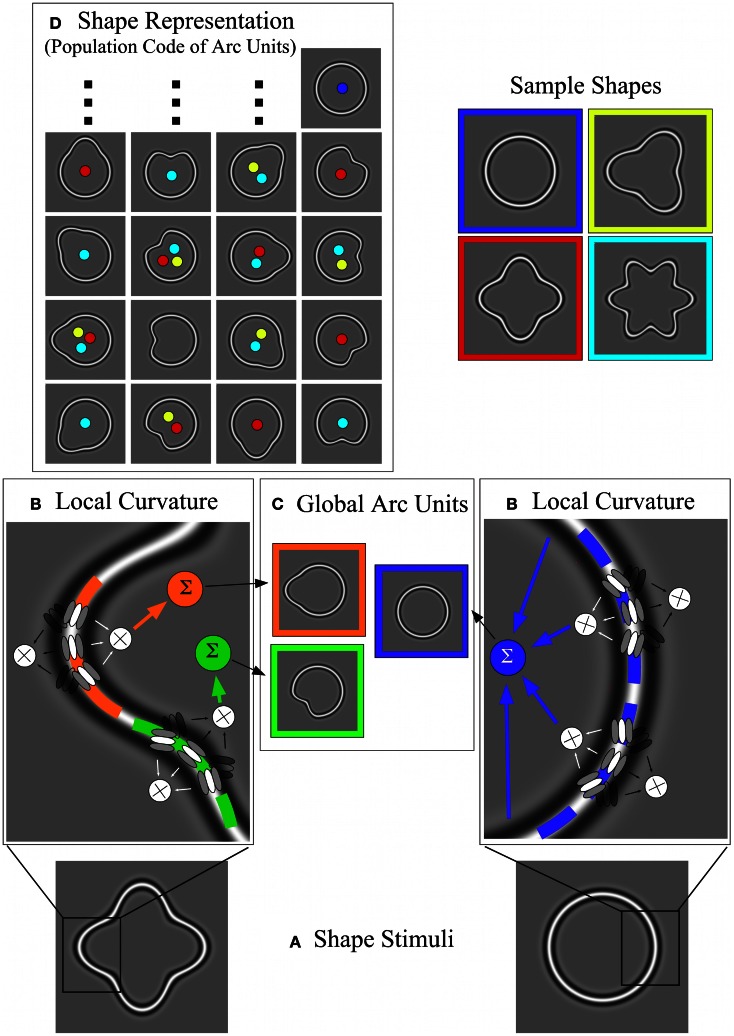
**Overview of the model including local curvature processing, arc units and population coding. (A)** Two sample shapes. Left: high-amplitude RF 4 with convex and concave points of curvature (at the peaks and troughs of the sinusoidal modulation). Points of convex and concave curvature are separated by inflection points with zero curvature. Right: a circle with uniform curvature throughout. **(B)** Local curvature processing. The images show details of the two sample shapes and superimposed triplets of V1 orientation filters used to extract local curvature (see also Figure 10). Curvature processing is supposed to be locally antagonistic (convex vs. concave); “active” triplets are shown in high contrast and their responses are combined multiplicatively. For the RF 4 (left), a “convex” triplet responds to the orange segment and a “concave” triplet to the green segment. For the circle (right), a number of “convex” triplets are active. **(C)** Global arc units. The responses of local curvature units **(B)** are integrated (Σ) along the contour up to the points where the contour's curvature changes sign. This integration is supposed to be multiplicative (see text). The local curvature responses are combined with information about the center of the contour and the distance from the contour to the center, which are computed in parallel by “global arc units.” These global arc units are therefore tuned to the location of a shape's points of local curvature extrema relative to its center (the relation to the center is symbolized by showing arc units as closed “shapes” with an obvious center), consistent with V4 physiology (Pasupathy and Connor, 2002). Depending on the length of the contour segments without changes in curvature sign (arcs), these global arc units receive input from one [high-amplitude RF4; **(B)** left] or many [circle; **(B)** right] local curvature units. For the examples shown **(C)**, one arc unit is sensitive to a convexity at 9 o'clock (orange), one responds to the concavity at 7 o'clock (green), another (blue) receives input from multiple local curvature units sampling the entire circumference of the circular contour [**(B)** right]. **(D)** Shows the shape representation as a population code of arc units. The first and third columns show arc units sensitive to convexities at various positions, whereas the second and fourth columns show arc units sensitive to concavities. Activation of units within this population code depends on the shape of the stimulus. Active arc units to sample shapes (blue = circle; yellow = RF3; red = RF4; turquoise = RF6) are shown by colored dots. For example, an RF 3 activates six arc units (yellow dots), an RF4 eight arc units, an RF 6 twelve arc units (only 10 of which are shown). A circle activates only one arc unit. Hence, shapes can be differentiated on the basis of the pattern of active arc units.

A key question for any model explaining our results is how to predict a decrease in thresholds while early noise clearly increases. A possible solution is that the increase in early noise might be made insignificant by a stronger late noise (Morgan et al., 1998; Parkes et al., 2001). To implement this idea, our modified model assumes a strong late noise source, possibly at the stage of area V4 that ultimately limits performance. Cells in area V4 exhibit tuning for curvature, distance from the shape center and polar angle of stimulus arcs. For modeling purposes we termed model filters with characteristics similar to those of V4 cells “arc units.” These arc units are different from local curvature units (Figure 10), which only extract the local curvature at various points along the contour. Arc units combine the responses of one or more local curvature units with inputs from earlier stages of the model, i.e., information about the shape center and the radial extent of the shape at different points (Poirier and Wilson, 2006). The resulting response of one arc unit provides a code of curvature at a certain polar angle with respect to the shape center. Finally, the responses of multiple arc units responding to a contour form a population code (Figure 11) for the representation of simple shapes (Pasupathy and Connor, 2002; Poirier and Wilson, 2006).

In addition to noisy arc units, a further important assumption of our model is that each arc of a shape is sampled by one or more local curvature units. For some shapes, one local curvature unit spans one relatively short arc, e.g., in an RF 4 or RF 8 shape. Longer arcs without changes in the sign of curvature, e.g., in the case of a full circle, necessitate the use of several local curvature units feeding into a single arc unit. The outputs of the local curvature units are combined multiplicatively, so that a diminished response in one local unit leads to a similarly diminished input to the entire arc unit. It is for these arc units that the noise is assumed to be *high* and *constant*. As a result, a circle, containing only one arc, is represented by a single arc unit, whereas high-amplitude RF 4 and RF 8 patterns are represented by a population of 8 and 16 arc units, respectively.

For incomplete RF 4 and RF 8 patterns (partial contours), as used in the spacing and set-size experiments, the number of active arc units depends on the number of segments without changes in the sign of curvature that are contained within the pattern. The stimuli used in the spacing and set-size experiments do not usually have an integer number of arcs, and, for simplicity, the number of arcs used for calculating the predictions of this model is not restricted to integer values, although, one might well-argue for “rounding to the nearest integer.” An increasing number of arcs, and, thus, an increasing number in noisy arc units, according to probability summation, leads to a decrease in performance, which is reflected in our experimental results. This causes the number of arc units to be the relevant or “effective” set-size for the prediction of performance in our heterogeneity detection task, rather than the number of Gabor patches.

#### Decision rule

The model assumes that performance for our task (detecting if one element is misaligned with respect to the contour) is determined by a population code of arc units analogous to cells in area V4 (Pasupathy and Connor, 2002; Poirier and Wilson, 2006), where individual arc units represent individual arcs. Any arc unit response will be maximal when the position and orientation of the Gabors are aligned with the subunits of the local curvature unit(s) feeding into the arc unit. The observers' task was to decide which of two patterns contained a misaligned element (orientation increment). For a complete RF 8, all of the activated 16 arc units, which represent individual arcs, respond optimally when the elements are aligned to the RF 8 contour (base pattern). For the increment pattern with one misaligned element, only 15 arc units respond optimally whereas one arc unit responds sub-optimally.

In visual search tasks, observers are often assumed to use a max decision rule when looking for a target among distractors, e.g., in a 2AFC with multiple distractors, they choose the interval that gives the highest response to any of the elements (Graham, 1989; Verghese, 2001). In our experiments, observers had to report the interval with an orientation misalignment, which results in a weakened response for the target. Therefore, in analogy to a max decision rule, we assume a min decision rule (i.e., looking for the minimum response in the hypothetical arc unit population). This is consistent with probability summation over arc unit responses (Graham, 1989; Verghese, 2001). Consider the example of an RF8 shape and a 2AFC task as used in our experiments. Two stimuli are presented to the observer (pattern A and pattern B). The subject's decision would be

*“Pattern A contains the misaligned element”* if min (A1, A2,…, A16) < min (B1, B2,…, B16), and *“Pattern B contains the misaligned element”* if min (A1, A2,…, A16) > min (B1, B2,…, B16),

where A1, A2, A3,…,A16 are the individual responses of the activated arc units to pattern A, and B1, B2, B3, …, B16 the responses to pattern B.

### Modulating factors

The following two factors, absolute curvature and inter-element spacing, were not part of the original model (Poirier and Wilson, 2006) but have to be considered here. As discrimination of isolated curvature arcs (segments without a change in sign of curvature) depends on average unsigned curvature (Bell et al., 2009b; Schmidtmann et al., 2012, 2013), and because the outputs of local curvature units feed the model's arc units, it is natural to assume that performance differs for shapes with different curvatures. The average unsigned curvature is the magnitude of a shape's average curvature ignoring its sign, i.e., if it is convex or concave.

In support of this, performance in perceptual tasks such as contour detection (Field et al., 1993; Pettet, 1999; Mathes and Fahle, 2007), curvature discrimination of curved segments (Wilson and Richards, 1989), curvature discrimination of texture boundaries (Wilson and Richards, 1992) and a probe comparison task (Barenholtz and Feldman, 2003) has been shown to deteriorate with increasing curvature. Sensitivity also decreases for RF pattern discrimination (Bell et al., 2009b; Schmidtmann et al., 2012) and detection (Schmidtmann et al., 2013) with increasing amplitude (and hence increasing curvature), although the effect of manipulating radial frequency varies across tasks. RF *detection* decreases with increasing frequency (Schmidtmann et al., 2013), whereas RF *discrimination* remains largely constant (Wilkinson et al., 1998). Consistent with Pettet (1999), who found in a contour detection task that “*the effect of changes in magnitude of curvature were predicted by the average of local curvature along the length of the contour*”, we used the average unsigned curvature (see supplementary material for equations) to modulate model sensitivity. The average unsigned curvature is highest for the RF 8 (*C*_av, unsig RF8_ = 1.1795), intermediate for the RF 4 (*C*_av, unsig RF4_ = 0.6033) and lowest for the circle (*C*_av, unsig circle_ = 0.37). Consistent with this, thresholds were typically highest for an RF 8 (*A* = 0.1) intermediate for an RF 4 (*A* = 0.18) and lowest for a circle.

Inter-element spacing, λ, also affects model predictions. This is based on observations of an improvement in performance for contour detection (Field et al., 1993) and for the detection of positional perturbations (Keeble and Hess, 1999; Levi and Klein, 2000) with decreasing spacing.

The overall model performance is therefore dependent upon the number of arcs in the shape (S), its average unsigned curvature (*C*_avg_) and the inter-element spacing between adjacent elements (λ). These parameters are dictated by the stimulus and are derived from theoretical considerations and are not the result of data fitting. Model predictions were based on Equation 3:

(3) $$T_{x}=T_{\text{circle}-\text{25}}\cdot S_{x}^{\;0.3}\cdot {\left(\frac{C_{\text{avg},x}}{C_{\text{circle}}}\right)}^{\text{​}1}\cdot {\left(\frac{\lambda_{x}}{\lambda_{\text{circle}-\text{25}}}\right)}^{\text{​}0.5}$$

*T*_x_ is the predicted threshold for experimental condition x. *T*_circle-25_ is the measured threshold (7.4°) for a circular pattern with 25 elements [measured once in the shape alignment experiment (*T* = 7.43°), and once again, albeit with different subjects, in the set-size experiment (*T* = 7.42°)]. This serves as a baseline relative to which all other thresholds are calculated. We chose the mean of subjects' thresholds for a 25-element circle as the baseline, since this is the most “basic” of our stimuli, and since inter-subject variability for this stimulus was very low. *S*_x_ refers to the number of activated arc units, which correlate with the number of arcs in the stimulus. The outputs of these arc units for each of the two patterns in a trial are compared employing a min decision rule. The dependence of thresholds on the number of active arc units is given by probability summation (Palmer et al., 2000). This predicts that log thresholds increase linearly with the log of the number of active arc units, showing a linear relationship in log-log coordinates, with a slope of about 0.3 (Palmer et al., 2000). The exponent (0.3) reflects this relationship. *C*_avg, x_ is the average unsigned curvature of the shape in condition *x*, which is normalized by the average unsigned curvature of the baseline condition *C*_circle_. The (redundant) exponent of 1 is based on the observation that the threshold for curvature discrimination increases with curvature with a log-log slope of 1 (Wilson and Richards, 1989). Finally, the inter-element spacing (λ_x_) of condition *x* is normalized by the inter-element spacing of the baseline condition (λ_circle-25_). The exponent of the last term was the only free parameter of the model. Its value (0.5) was calculated (method of least squares) to give the best fit to the experimental data.

We did not explicitly apply the model described by Poirier and Wilson with our proposed elaborations and did not calculate its direct response to our stimuli. Full model simulations were beyond the scope of this study. Instead, we focused our analysis on how the model response would differ for various parameter manipulations. In this sense, the “basic Poirier and Wilson model” output is assumed to be given by our baseline condition (*T*_circle-25_) and the terms in Equation 3 are to reflect how the model response would change depending on various parameter manipulations.

### Model predictions

**Figure 12:**
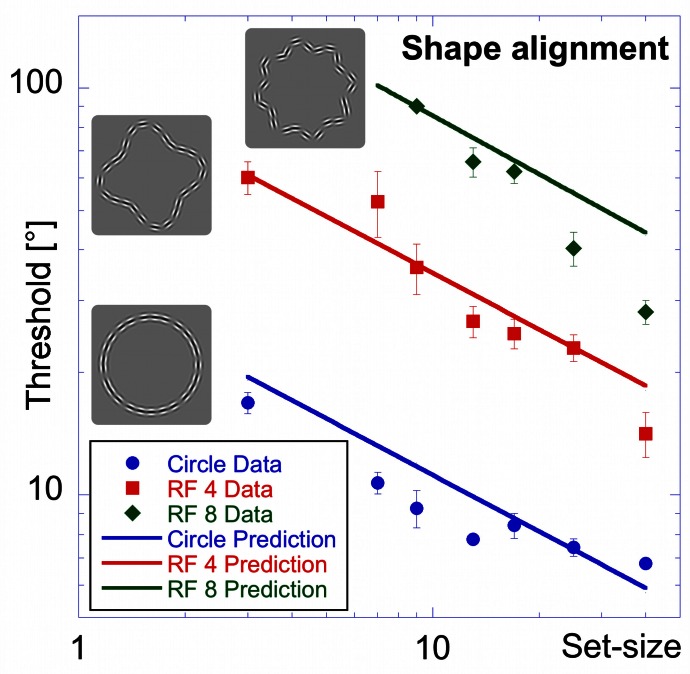
**Model predictions vs. experimental data—shape alignment experiment.** Here, and in Figures 13 and 14 blue circles, red squares and green diamonds represent experimental data for circle, RF 4 and RF 8, whereas the colored lines show the respective model predictions.

**Figure 13:**
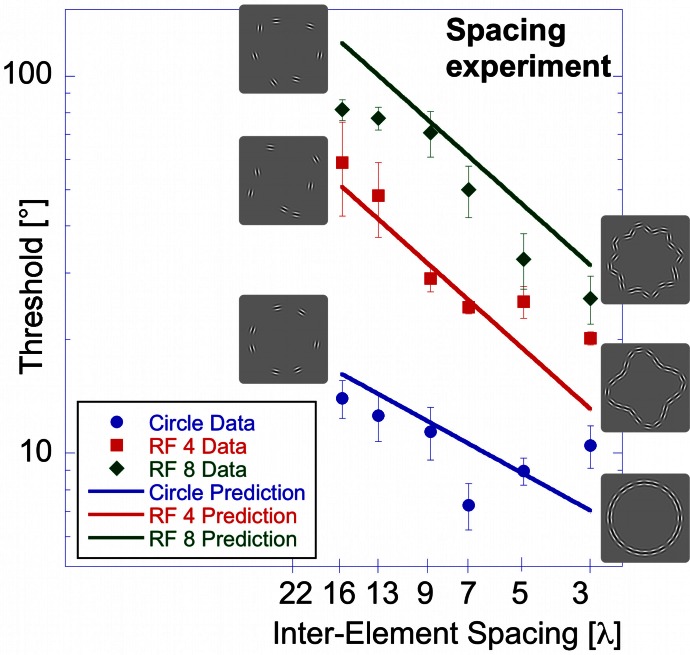
**Model predictions vs. experimental data—spacing experiment**.

**Figure 14:**
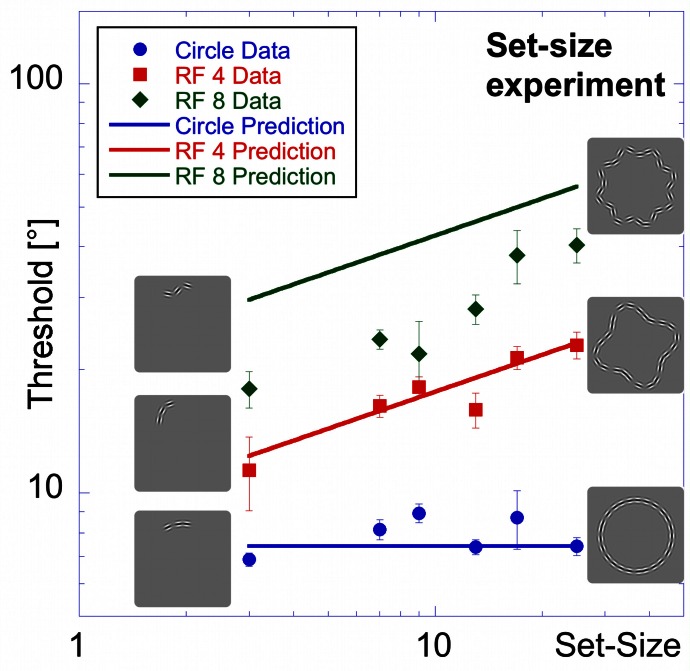
**Model predictions vs. experimental data—set-size experiment**.

Figures 12–14 show the model predictions (blue line for RF 0; red line for RF 4; green line for RF 8) and the experimental data (blue circles for RF 0; red squares for RF 4 and green diamonds for RF 8). For the shape alignment experiment (Figure 12), the model captures the data well. As can be seen from Equation 3, the dependence of thresholds on set-size is the same for all shapes and therefore gives lines parallel to each other. Differences between shapes (vertical shifts of the curves) are due to differences in the number of activated arc units and shape curvature.

Figure 13 shows the model prediction for the spacing experiment. The effect of inter-element spacing is also generally well-captured by the model. For the RF 8, the slope of the model prediction line (how sensitivity depends on spacing) is appropriate (this follows from Equation 3) although the model slightly under-estimates observer sensitivity. The prediction for the circle remains unaltered compared to the shape alignment experiment. Steeper slopes for the RF 4 and RF 8 result because with increasing spacing, the number of activated arc units increases for these shapes, compared to the circle, where only one arc unit is active irrespective of the length of the sampled circular contour.

Figure 14 shows thresholds and predictions as a function of set-size for the set-size experiment. The model provides an excellent fit for both circular and RF 4 data. As for the effect of inter-element spacing, the predictions for circular and RF 4 data are good, as is the slope for the RF 8, while absolute thresholds for the RF 8 condition are, however, overestimated. The elevation of predicted thresholds with increasing RF stems mostly from the different average curvatures (Equation 3) because spacing is constant for all shapes and set-sizes. On the other hand, the slopes of the model predictions depend on the number of arcs, which increases with set-size for RFs 4 and 8. Given the exponent in Equation 3, the predicted slope is 0.3. For the circular shape, the slope is zero, because the number of active arc units is constant (one) irrespective of the length of the sampled contour.

In summary, a wide range of experimental data is generally well-captured by the model, which is based on physiologically plausible mechanisms.

## Discussion

### Implications of experimental data for visual search

Current decision noise models of visual search (Palmer, 1994, 1995; Palmer et al., 2000) do not generally differentiate between uniform and random configurations and predict the same set-size slopes (in the range of +0.20 to +0.35). The set-size effect observed in the random orientation experiment is substantially higher (slope = +1.1). This result is well-captured by limited capacity models (Treisman and Gelade, 1980; Palmer et al., 2000), which predict slopes of +1, but which fail to predict slopes for aligned or parallel patterns.

It seems that randomizing element orientation might be a universal method to degrade performance relative to aligned or parallel configurations in various visual tasks, perhaps because the many different orientations do not allow adjacent elements to be bound or grouped (Duncan and Humphreys, 1992) by local or global processes.

In their orientation discrimination experiments, Palmer (Palmer et al., 1993; Palmer, 1994) and Pavel (Pavel et al., 1992) found considerably smaller slopes of +0.23, and +0.37 than for our random condition. Adding distractor heterogeneity (a small amount of orientation jitter) resulted in a threshold elevation for all set-sizes, but no considerable change in set-size slope (+0.37 vs. +0.4; Palmer et al., 2000). Palmer et al. (2000) suggested that these small slopes (small compared to those predicted by limited capacity models) argue in favor of a decision noise model rather than a limited capacity model. The results of our uniform (zero set-size slope) and random condition (slope = +1.1) put this hypothesis into question. It is possible that when distractors are of uniform orientation or when the distractor heterogeneity is small, the elements can be grouped, thereby diminishing the effect of set-size on performance (Duncan and Humphreys, 1989; Kingstone and Bischof, 1999; Roggeveen et al., 2004). In this case, the small slopes found by Palmer might not be due to a decision noise mechanism, but, instead, due to a limited capacity mechanism where distractors can be grouped. However, a quantitative grouping theory of the same predictive power as the decision noise or limited capacity model has yet to be developed. The uniform orientation experiment (Figure 2) showed that the detection of an orientation increment in a uniform pattern is independent of the number of elements in the display, confirming results from earlier studies (Orbach et al., 2005). The zero set-size slope in the uniform orientation experiment is not predicted by any of the models of visual search mentioned in the introduction. One might argue, however, that the absence of a substantial set-size effect is a classic example of pop-out, indicating a parallel search (Treisman and Gelade, 1980). However, Palmer and colleagues (Palmer and Mclean, 1995) argued that such pop-out is only observed when stimuli are presented well above threshold, and that pop-out might be an artifact of not carefully calibrated stimuli. The argument was that, if the stimuli in the so-called classical pop-out tasks were presented near threshold, then one does not see pop-out, but a regular set-size effect. Following this line of argument for our threshold stimuli, a zero set-size slope is indeed surprising. It remains unclear, however, why the earlier orientation discrimination experiments (Pavel et al., 1992; Palmer et al., 2000) gave a non-zero set-size slope for parallel distractors, opposed to the results of our “uniform” orientation experiment.

The improvement in performance that is sometimes seen as set size increases—or any performance better than predicted by signal detection theory models—might also be argued to be related to the configuration superiority effect (Pomerantz et al., 1977; Bacon and Egeth, 1991) or to attentional engagement theory (Duncan and Humphreys, 1992). According to this, the relationship between the elements, e.g., their parallel orientation, allows distractors to be grouped, and thus affects orientation discrimination performance, resulting in a substantially higher sensitivity for the uniform condition than for the random orientation condition. We certainly see an effect of the configuration improving performance. However, to establish a connection between our inverse set size effect with the classic configuration superiority effect, further experiments are required to systematically evaluate the effect of set size on stimuli, generalizing those used for the classic effect and also to use threshold, rather than suprathreshold discrimination.

One possible reason for the difference between random and uniform orientation patterns is that, for certain arrangements, mechanisms tuned to global stimulus properties (e.g., texture or extended contour shape) become engaged and their performance exceeds that of the process responding to random arrangements including those typically seen in visual search. This hypothesis was confirmed by the shape-alignment experiment. Consistent with the uniform condition, if elements are aligned tangent to a contour, the detection of an orientation increment is not adversely affected by increasing the numbers of elements. In contrast to the uniform condition, performance, rather than being unaffected, actually improves as the number of elements increases. Such an improvement with increasing set-size is surprising when viewed in the context of visual search because an increase in set-size is accompanied by an increase in spatial uncertainty as to the number of possible positions where the increment patch might appear. According to current theories of visual search, having to spread attention over a larger number of positions, or having to monitor more orientation detectors (Palmer et al., 2000), should negatively affect sensitivity. The results of the shape alignment experiment are, however, in qualitative accord with a number of investigations on shape perception (Braun, 1999; Keeble and Hess, 1999; Levi and Klein, 2000; Li and Gilbert, 2002; Mathes and Fahle, 2007) who found performance for contour detection in noise (or, in the case of Keeble and Hess, performance for detection of position displacement from a contour) to improve with an increasing number of contour elements and/or with decreasing inter-element spacing. This discrepancy may be explained in the following way: in a visual search task where multiple elements that are uncorrelated (i.e., not forming a shape or a texture) have to be monitored, observer performance is captured by a “visual search” process and declines with set-size. Irrespective of the exact nature of the visual search mechanism (a serial mechanism or a spatial uncertainty mechanism, Palmer et al., 2000), it results in a positive set-size effect. Such a search mechanism would presumably always be available when confronted with multiple elements. However, as soon as the elements are arranged in such a way that they stimulate other mechanisms (when pattern elements become part of a larger context, e.g., form a texture or a shape), the response from both mechanisms would be available and observer performance would be limited by whichever one is more sensitive. Visual search processes with low sensitivity only predict performance when the stimulus is deprived of any global context. For example, for the shapes used in the “shape alignment” experiment, a classical visual search mechanism monitoring multiple elements with heterogeneous orientations, would predict a positive set-size slope. A shape mechanism, on the other hand, would explain the negative set-size slopes because it is more sensitive to orientation changes of individual elements forming a shape. The reason why thresholds decrease with increasing set-size might then be due to a finer sampling advantage enabling the shape mechanism to make more accurate discriminations.

### Implications of experimental data for shape processing

#### Effect of set-size and spacing

The similarity of the results for equivalently sampled contours (shape alignment experiment) and partial contours (spacing experiment) indicates that the main factor driving performance is inter-element spacing, not set-size. Narrow spacing improves processing of the contours and yields higher sensitivity for the detection of an orientation deviation of one element. This is in agreement with Levi and Klein's (2000) results for a task of detecting positional jitter of pattern elements within sampled circles.

Performance for the random orientation experiment, however, indicates that this is not a general effect. Spacing has no, or very little, influence on the processing of that type of element arrangement. For patterns with randomly oriented elements, the main factor determining performance is set-size.

Furthermore, if spacing alone was the determining factor for the detection of orientation heterogeneities, the set-size experiment, where set-size was varied while separation remained fixed, should have produced flat set-size curves for all shapes. This was indeed found for circular patterns, but not for RF 4 and RF 8. Hence, set-size and spacing have qualitatively and quantitatively different effects on performance depending on shape. For the circle, varying set-size while keeping spacing constant does not alter performance. For RF 4 and RF 8, on the other hand, an increase in set-size, with constant spacing has a detrimental effect. For all shapes, decreasing spacing while keeping set-size constant improves performance. When the two effects co-occur (increase in set-size and a decrease in spacing; “shape alignment” experiment), spacing dominates. The dominant role of spacing is consistent with earlier reports on circles (Levi and Klein, 2000), but the results for the RF 4 and RF 8 shapes point toward more complicated interactions between set-size and spacing for non-circular shapes.

#### Effect of stimulus shape

The other main finding of the experiments is that the stimulus shape strongly affects thresholds. The “shape alignment”, “spacing” and “set-size” experiments deliver converging evidence that sensitivity for the detection of orientation increments decreases with increasing radial frequency. This result is in accord with a recent study showing that thresholds for the detection of RF patterns embedded in noise increase with radial frequency (Schmidtmann et al., 2013). Decreasing performance with increasing radial frequency might also be indicative of a shift from global processing to local computations, which has been reported for radial frequencies of about 8–10. This shift has been shown for low (Jeffrey et al., 2002; Loffler et al., 2003) and high (Schmidtmann et al., 2012) amplitudes.

On the other hand, studies investigating RF discrimination from a circle have shown that there are only insignificant perceptual differences between different RF shapes (Wilkinson et al., 1998), yielding constant discrimination thresholds for different RFs.

Consequently, depending on the task (on one hand detection of orientation increments in RF shapes and RF detection, on the other hand RF discrimination), performance does or does not decrease with increasing RF.

### The model

The Poirier and Wilson model was developed for, and applied to, continuous RF patterns. It is unclear how it would respond to sampled RFs. Sampling a contour would have an effect on a number of model components: obviously local curvature but also likely the extraction of the center of the contour and its radial extent. Since all of these feed into subsequent stages and since many stages are non-linear, it is difficult to predict the model's behavior to sampling. As our data show, sampling has a profound effect on sensitivity, but its effect also depends on shape: for RF 4 and RF 8 shapes, narrow spacing improves performance, for circles there is no effect of spacing. Furthermore, it is not clear how the Poirier and Wilson model would respond to our task of heterogeneity detection. Orientation increments that appear in concave arcs would probably not be detected, whereas increments in convex arcs might lead to a diminished response of a local curvature unit sampling that arc, or it might result in responses from curvature units tuned for higher or lower curvature than that of the arc.

However, making a number of modifications (based on our and other laboratories' data) to the original Poirier and Wilson model which still retained its basic spirit of a population code to represent shape and incorporating well-established psychophysical results, we were able to derive an equation (Equation 3) that we would expect to be consistent with detailed mathematical modeling from this, or, indeed, a whole class of models for shape perception. Such models have the following properties: Firstly, the models assume performance in shape tasks to be based on a population code of arc units which respond to both convexities and, when present, concavities, in agreement with psychophysical work (Pettet, 1999) and that these arc units essentially signal the arcs (segments without change is the sign of curvature) contained within a shape. Secondly, we assume that performance is limited by late noise (Morgan et al., 1998; Parkes et al., 2001) in these units. Thirdly, the average unsigned curvature of the RF pattern modulates sensitivity, in agreement with earlier work (Pettet, 1999). This last point seems reasonable as curvature discrimination, within a curvature range of 1/deg to 10/deg, follows a Weber law relationship (Wilson and Richards, 1989). To reflect this, we incorporated a modulating factor depending on the average curvature of the contour. This has the effect of uniformly elevating thresholds for shapes with higher average curvature. Note that in the curvature factor, average unsigned curvature of the RF pattern is normalized by the curvature of the unmodulated circle, so that the RF radius is cancelled out (see supplementary material for curvature equations). Therefore, thresholds predicted by our model are scale invariant, consistent with other studies (Wilkinson et al., 1998). Fourthly, inter-element spacing has been shown to have a strong influence on contour integration contour detection tasks (Field et al., 1993; Levi and Klein, 2000) as well as contour appearance (Day and Loffler, 2009). Inter-element spacing was incorporated into the model and elevates thresholds with increasing spacing.

The Poirier and Wilson model uses a population code as a representation of simple shapes and the model's sensitivity is based on that. That model, applied to the discrimination of continuous RF patterns, assumes a cross-correlation of a given pattern with an internal template, analogous to Edelman's representation of similarities (Edelman, 1998). The discriminability between two shapes (pattern A vs. pattern B) is defined as the difference between the two cross-correlations, i.e., the distance in a multi-dimensional shape space representation. Our model assumes a min decision rule comparing the population code responses of the bank of arc units to patterns A and B. The Poirier and Wilson model may have certain advantages for efficiency of coding and for the invariant recognition of shape, but we chose the min rule using the original population codes for patterns A and B as an algorithm arising naturally out of signal detection theory.

## Conclusions

The aim of the experiments presented here was to determine the effects of set-size, shape, spacing and other factors on the detection of contour heterogeneity, and also to put our results into context with existing models of visual search and shape processing.

Pattern shape has a strong effect on performance, which deteriorates with increasing complexity (radial frequency). Dense sampling improves performance for patterns with elements aligned to the contour, but not for random orientation patterns. We found high set-size slopes, consistent with the prediction of limited capacity models, for randomly oriented elements, a zero set-size slope for parallel elements, and negative set-size slopes when elements were aligned to a contour shape. This is consistent with the proposal that whenever elements cannot be grouped (when they are, e.g., randomly oriented), performance is limited by a process that monitors multiple elements, similar to the bulk of experiments on visual search. However, when the configuration supports grouping (in the form of texture or global contours) performance is limited by more sensitive, global pooling mechanisms. For the parallel configuration, the zero set-size slope may be explained by a global mechanism for parallel texture, performance of which has been shown to be independent of the number of elements and of element density in the display (Wilson and Wilkinson, 1998). For the experiments where elements were aligned to a contour performance is well-explained by the model presented in this paper.

A sensible generalization of our results for random, uniform and aligned shapes is that the “effective set-size” determines performance for heterogeneity detection, where the effective set-size is equal to the number of pattern parts that can be grouped. For patterns with random Gabors, the effective set-size is equal to the number of Gabor patches because grouping does not take place. For aligned patterns, the effective set-size is equal to the number of segments without a change in the sign of curvature (arcs), and performance for these patterns is also influenced by spacing and curvature. In the uniform orientation patterns, the effective set-size is equal to one, because all elements can be grouped into one texture. A prediction from this is that, for texture patterns containing more than one orientation, the effective set-size is equal to the number of “interleaved textures.”

The results of our experiments provide insight into the relation between contour integration, shape and texture mechanisms. When elements were aligned to a shape, performance was better than when they were randomly oriented. This, and the effect of spacing on performance, supports the assertion by Wilkinson et al. (1998) that an association field or collinearity mechanism (Field et al., 1993; Polat and Sagi, 1993) enhances the input into a shape mechanism. Under the assumption that the uniform condition probes texture processes whereas the aligned conditions probe global shape processes, differences between the conditions can be attributed to differences for the two types of global computations. The uniform orientation (texture) results show a zero set-size slope, whereas the aligned circle (shape) results show a negative slope. This indicates that, although the sensitivity of a mechanism for circular shape and a mechanism for parallel texture seems to be similar, increased set-size and proximity improves performance for shape, but increased set-size and higher density of elements does not improve performance for parallel texture, in agreement with reports on Glass patterns (Wilson et al., 1997; Wilson and Wilkinson, 1998).

The model presented here is based on physiologically plausible mechanisms, and the equation to predict performance is derived from stimulus properties, such as spacing, curvature, and complexity of the shape (RF). With only one free parameter the model fits our experimental data well. It is based on the assumption of late noise, i.e., on the testable possibility that the noise in arc units (the model equivalent of V4 cells) is higher than the noise in orientation filters (equivalent to V1 cells). Although psychophysicists have previously suggested late noise as an explanation for their experimental data (Morgan et al., 1998; Parkes et al., 2001), it would be useful to have an explicit experimental comparison of neurophysiologically measured noise in V1 vs. V4 cells.

### Conflict of interest statement

The authors declare that the research was conducted in the absence of any commercial or financial relationships that could be construed as a potential conflict of interest.
