# Supplementary material for: Set-size effects for sampled shapes: experiments and model
Source: Front Comput Neurosci. 2013 May 28;7:67. doi: 10.3389/fncom.2013.00067 (PMC3664879; doi:10.3389/fncom.2013.00067)
Supplement: Supplementary file 1 [file Presentation_1.PDF]

Supplementary material

Equations for the calculation of the model predictions

1. Number of arcs

The number of arcs (S, contour segments without a change in sign of curvature) for RF patterns containing concavities is given by Equations A1 – A3.

Shape alignment experiment: $S = 2 \cdot RF$ Equation A1

where RF is the radial frequency

Spacing experiment: $S = S_{\max} \cdot \frac{n}{n_{complete}} = 2 \cdot RF \cdot \frac{7}{n_{complete}}$ Equation A2

where S_{max} is the number of arcs of a complete RF pattern given by Equation A1, n is the set-size of the stimulus (here, n = 7), and $n_{complete}$ is the set-size that would be required, at each spacing, to sample a complete contour.

Set-size experiment: $S = S_{\max} \cdot \frac{n}{n_{\max}} = 2 \cdot RF \cdot \frac{n}{25}$ Equation A3

where S_{max} is the maximum number of arcs ($S_{max,RF4}$ = 8; $S_{max,RF8}$ = 16), and n_{max} is the maximum number of elements (here, n_{max} = 25) fitting on the contour to produce a closed contour.

2. Average unsigned curvature

For an arbitrary curve $\rho = f(\varphi)$,

$$K = \frac{\rho^2 + 2 \cdot \rho'^2 - \rho \cdot \rho''}{\left(\rho^2 + \rho'^2\right)^{\frac{3}{2}}}$$ Equation A4

K is curvature of the arbitrary curve $\rho = f(\varphi)$, φ is the polar angle and ρ' and ρ'' are respectively the first and second derivative of ρ with respect to φ (Bronstein et al., 1995).

For an RF pattern, curvature $C(\varphi)$ as a function of the polar angle φ is therefore given by Equation A5,

$$C(\varphi) = \frac{\left(R(\varphi)\right)^2 + 2 \cdot \left(R'(\varphi)\right)^2 - R(\varphi) \cdot R''(\varphi)}{\left(\left(R(\varphi)\right)^2 + \left(R'(\varphi)\right)^2\right)^{\frac{3}{2}}}$$ Equation A5

where $R(\varphi)$ is the function describing an RF curve, given in Equation A6.

$$R(\varphi) = r \cdot \left(1 + A \cdot \sin\left(RF \cdot \varphi + \theta\right)\right)$$ Equation A6 (Wilkinson et al., 1998)

where r is the radius of the unmodulated circle, A is the amplitude of radial modulation, RF is the radial frequency, and θ is the RF pattern phase.

$$R'(\varphi) = r \cdot A \cdot RF \cdot \cos(RF \cdot \varphi + \theta)$$ Equation A7

$$R''(\varphi) = -r \cdot A \cdot RF^2 \cdot \sin(RF \cdot \varphi + \theta)$$ Equation A8

The average unsigned curvature is equal to the integral over one cycle of the RF pattern (φ = 0 to 2π/RF) of the absolute values of the curvature function, *C(φ)*, divided by the arc length of the first cycle, $\frac{2\pi}{RF}$.

$$C_{av,unsig} = \frac{\int_0^{\frac{2\pi}{RF}} |C(\varphi)| d\varphi}{\frac{2\pi}{RF}}$$ Equation A9

References

Bronstein, I.N., Semendjajew, K.A., Musiol, G., and Mühlig, H. (1995). *Taschenbuch der Mathematik.* Thun und Frankfurt am Main: Verlag Harri Deutsch.

Wilkinson, F., Wilson, H.R., and Habak, C. (1998). Detection and recognition of radial frequency patterns. *Vision Research* 38, 3555-3568.
